# Clinical heterogeneity and prognostic determinants in rheumatoid vasculitis: a systematic analysis of organ-specific manifestations, therapeutic outcomes, and biomarker correlations

**DOI:** 10.3389/fimmu.2025.1628504

**Published:** 2025-09-15

**Authors:** Dongyi Wang, Le Lu, Junyi Shen, Yuping Zhang, Muzi Li, Wei Shang

**Affiliations:** ^1^ Department of Integrative Medicine, Jinling Hospital, Affiliated Hospital of Medical School, Nanjing University, Nanjing, China; ^2^ Postdoctoral Research Station, Jinling Hospital, Affiliated Hospital of Medical School, Nanjing University, Nanjing, China

**Keywords:** rheumatoid vasculitis, biologic DMARDs, anti-CCP antibodies, multi-organ involvement, prognostic biomarkers, infection risk stratification

## Abstract

**Background:**

Rheumatoid vasculitis (RV), a severe extra-articular complication of rheumatoid arthritis (RA), with current evidence limited to fragmented case reports and a lack of consensus on diagnostic/therapeutic protocols. This study systematically evaluated the clinical heterogeneity, prognostic determinants, and management challenges of RV through the comprehensive analysis of case reports.

**Methods:**

We conducted a systematic review of 112 RV cases from PubMed, Scopus, and Embase in the past 10 years. After dual-reviewer screening and exclusion of confounded cases (concurrent autoimmune diseases, drug-induced vasculitis, infection-associated cases), data spanning demographics, organ involvement patterns, treatments, and outcomes were extracted. Subsequent statistical analyses and data visualization were conducted using R software.

**Results:**

The mortality group exhibited significantly higher rates of concurrent infections (*P* = 0.03) and elevated anti-cyclic citrullinated peptide (anti-CCP) antibody titers (*P* = 0.04) compared to the survival group. Only less than half of the medical records had histopathological confirmation of the diagnosis, which was particularly notable in cardiac (14.3%, 1/7), pulmonary (33.3%, 2/6),and cerebral (25.0%, 9/36) involvement. During the induction of disease remission, glucocorticoids remained the primary therapy. Both conventional synthetic disease-modifying antirheumatic drugs (DMARDs) (*P* < 0.05) and biologic DMARDs (*P* < 0.001) demonstrated favorable prognostic associations.

**Conclusions:**

This study highlighted RV’s heterogeneous organ involvement and underscored the prognostic value of anti-CCP and infection screening. The limited histopathological confirmation rates emphasized the need for multimodal diagnostics. Our findings also provided robust evidence supporting the therapeutic efficacy of biologic DMARDs in RV management.

## 1 Introduction

Rheumatoid arthritis (RA) is a systemic autoimmune disorder characterized by chronic joint inflammation, with extra-articular manifestations affecting multiple organ systems. Among these, rheumatoid vasculitis (RV)—a severe complication of RA—is characterized by inflammation of small-to-medium-sized blood vessels, leading to cutaneous, neurological, cardiac, pulmonary, and renal damage (1). RV carries significant morbidity and mortality due to its propensity for tissue ischemia and organ dysfunction, requiring timely diagnosis and treatment. Nevertheless, there is a striking absence of clinical guidelines for management and treatment. Systematic investigations into RV’s epidemiological patterns, heterogeneity in clinical manifestations, therapeutic strategies, and prognostic determinants remain limited. Current literature predominantly focuses on isolated case reports and small-scale cohort studies, which lack robust multivariate analyses. Consequently, more studies on systematic analyses of these RV cases are critically needed to establish evidence-based frameworks for managing this high-risk RA complication.

This study systematically analyzed clinical data from 112 RV cases derived from global case reports to comprehensively delineate demographic characteristics, clinical manifestations, and organ involvement, while analyzing diagnostic and therapeutic modalities. Furthermore, we investigated the critical factors influencing clinical outcomes to provide a foundation for optimizing early recognition, active interventions, and prognostic evaluation in RV.

## 2 Method

### 2.1 Data collection

We systematically searched the PubMed, Scopus, and Embase databases to identify studies published within the preceding decade on March 20, 2025, using the search terms: (“rheumatoid vasculitis”) OR (“rheumatoid arthritis” AND “vasculitis”). Given the paucity of large-scale studies on RV, clinical cases were chosen to extract granular clinical details. Our data sources encompassed conference abstracts, letters to the editor, case reports, and case series with enriched clinical narratives. After importing retrieved records into EndNote X8 bibliographic and reference manager, duplicates were manually identified and removed. If duplicate records exist across the three databases, PubMed-sourced articles were preferentially retained, followed by Scopus -derived articles. Initial screening was performed by two independent authors based on titles and abstracts to select case reports meeting preliminary eligibility criteria. The diagnosis was based on the 1984 diagnostic criteria proposed by Scott and Bacon (2), and RV was explicitly confirmed in the article. Full texts of these articles were then independently reviewed by both authors to confirm the inclusion.

Exclusion criteria encompassed: (1) Comorbidity with other autoimmune diseases (e.g., systemic lupus erythematosus, ANCA-associated vasculitis, IgA vasculitis) that may lead to the vasculitis; (2) drug-induced vasculitis (e.g., adalimumab, tocilizumab, golimumab); (3) vasculitis secondary to viral infections (e.g., Epstein-Barr virus (3)); (4) unavailable full text; and (5) non-RV cases. Discrepancies between reviewers during screening or data extraction were resolved through consensus discussions or adjudication by a third senior investigator. This rigorous dual-review process ensured methodological consistency and minimized selection bias in case inclusion.

### 2.2 Data categories

We systematically extracted data across 12 distinct categories: (1) demographic characteristics (gender, age); (2) year of publication, (3) author’s origin country, (4) clinical history (RA disease duration, RA disease activity, underlying medical condition, pre-onset treatment); (5) site of involvement; (6) clinical manifestations; (7) laboratory findings (rheumatoid factor [RF], anti-cyclic citrullinated peptide [anti-CCP], C-reactive protein [CRP], antinuclear antibody [ANA]); (8) histopathological finding; (9) co-infection status; (10) diagnostic approaches; (11) therapeutic interventions (pre-confirmation management, induction therapy, and maintenance regimens); and (12) outcomes.

### 2.3 Quality assessments

The quality of included cases was assessed by two authors using the Joanna Briggs Institute (JBI) critical appraisal tools, with quality scores ranging from 0 to 8 points. The standardized checklist for case reports was obtained from the official JBI resource website (https://jbi.global/critical-appraisal-tools) and implemented following the institute’s recommended appraisal procedures.

### 2.4 Data encoding

Some collected data underwent manual coding for data processing in R software (version 4.2.3), with variables assigned numerical values as follows: gender (male = 1, female = 2), pre-onset non-standardized treatment (yes = 1, no = 0), underlying medical condition (yes = 1, no = 0), RA disease activity (active=1, remission=0), concurrent infections during disease progression (present = 1, absent = 0), histopathological confirmation (available = 1, unavailable = 0), serum ANA test (positive = 1, negative = 0), and organ involvement (single-organ = 0, multi-organ = 1). Outcomes were classified as survival(1) and mortality (0), with NA assigned to lost follow-up cases. All undocumented variables were uniformly coded as NA, ensuring systematic handling of missing data during statistical modeling.

### 2.5 Data analysis

Statistical analysis and data visualization were performed using R software (version 4.2.3). Dichotomous variables were analyzed with either the Chi-square test or Fisher’s exact test, as appropriate for sample size requirements, while continuous variables were evaluated using the Mann-Whitney U test for non-normally distributed data. Statistical significance was defined as a two-tailed *P*-value < 0.05. Sites of RV involvement were anatomically mapped using the gganatogram package. Multi-organ involvement overlaps were visualized through a Venn diagram. Diagnostic methodologies were comparatively displayed in polar bar plots generated via the circlize package. Therapeutic regimens were stratified into three clinical phases: pre-diagnosis management, induction therapy, and maintenance treatment, visualized as stacked bar plots using ggplot2, and medication utilization frequencies were comparatively visualized using Cleveland dot plots. Correlation matrices derived from Spearman’s rank-order tests were represented as a heatmap.

## 3 Result

### 3.1 Screening results

**Figure 1:**
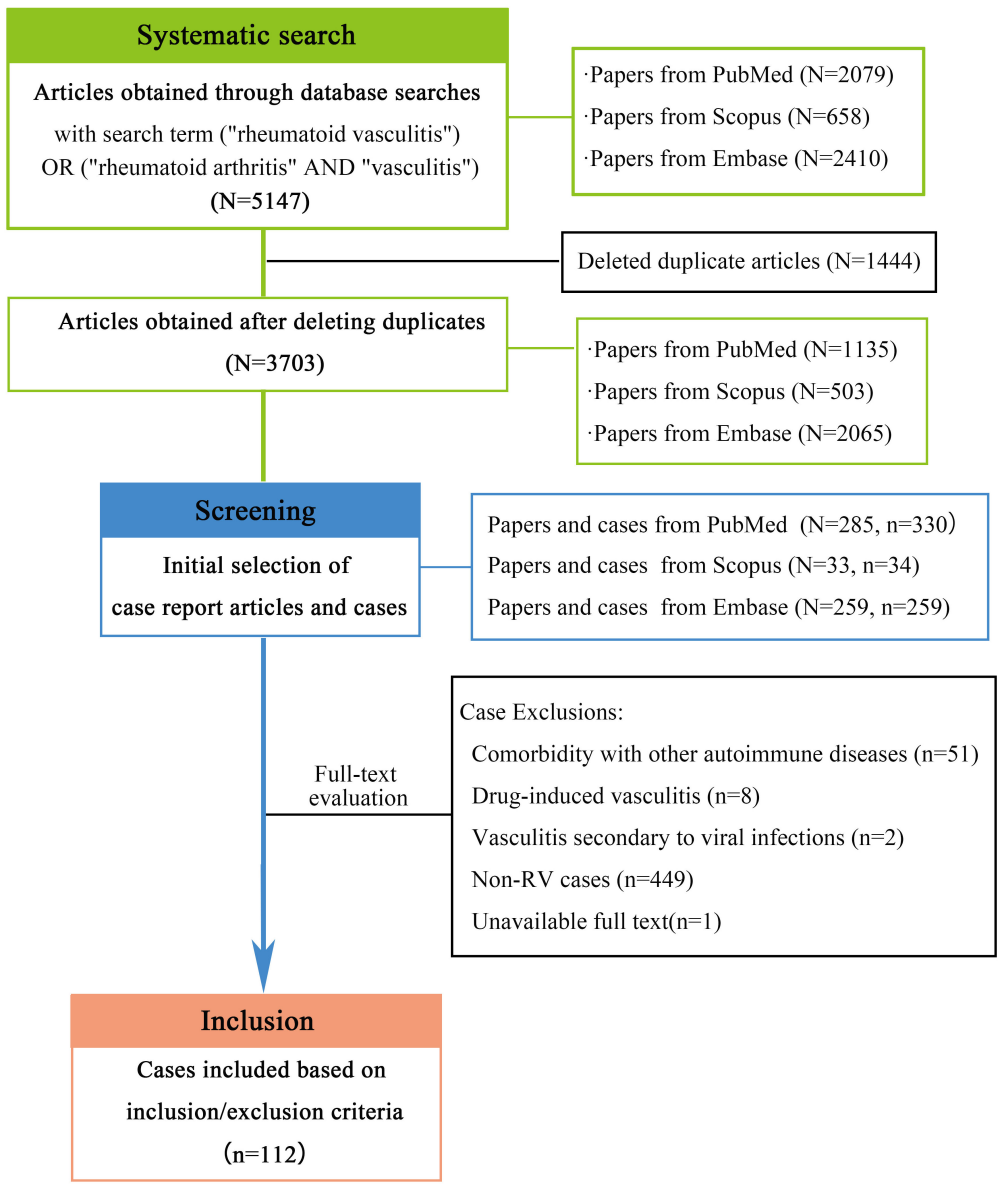
Literature search flowchart of RV clinical cases across three medical databases.

As shown in **Figure 1**,through a comprehensive literature search, we initially identified 5147 articles. After removing 1444 duplicates using EndNote, 3703 unique publications underwent title/abstract screening, from which 577 case report articles (encompassing 623 cases) were selected. Two authors independently performed full-text evaluations, systematically excluding cases based on predefined criteria: 449 out of topic; 51 involving coexisting immune disorders (including ANCA-associated vasculitis, systemic lupus erythematosus, IgA vasculitis, and systemic sclerosis); 8 drug-induced cases linked to biologics (including certolizumab pegol, tocilizumab, adalimumab, and golimumab); 2 virus-associated vasculitis; and 1 unavailable full-text article. Following this rigorous exclusion process, 112 clinically pertinent cases were retained for final analysis. The evaluation of medical records using the JBI criteria revealed the following distribution of scores: 7 cases scored 8 points, 73cases scored 7 points, 30cases scored 6 points, 1 case scored 5 points, and only one case scored 4 points.

### 3.2 Demographic and clinical characteristics

Analysis of the 112 included cases revealed 98 patients (87.5%) with improved outcomes, 12 fatalities (10.7%), and 2 cases lacking clinical outcomes (**Table 1**). The cohort demonstrated a median age of 60 years (IQR 51–71; range 6–89), with no distinct age-related disease pattern: 45 patients (40.2%) were ≥65 years versus 64(57.1%) <65 years. RV onset occurred across disease stages, ranging from initial RA presentation to cases developing after 57 years of RA disease duration. The cohort demonstrated a female predominance (73 females vs. 36 males), with a female-to-male ratio approaching 2:1. Comorbid conditions were relatively uncommon (14.3%, 16/112), with hypertension and type 2 diabetes being most prevalent. Nearly one-quarter of RV patients (22.3%, 25/112) exhibited active RA. Concurrent infections occurred in 14 patients (12.5%), including 4 bacteremia cases (28.6% of infections). Single-organ involvement predominated in RV (79.5% vs. 20.5% multi-organ). Most patients (82.1%, 92/112) received conventional RA therapy pre-onset, while 20 (17.9%) had discontinued or never initiated treatment. Histologic findings were limited (42.9%, 48/112), particularly for cardiac, pulmonary, and cerebral involvement due to the practical difficulties in obtaining biopsy specimens from critical organ systems. Notably, concurrent infections and anti-CCP antibody levels demonstrated prognostic relevance in RV. Mortality cases exhibited a markedly higher infection rate (33.3%, 4/12) compared to the improved-outcome cohort (9.2%, 9/98; *P* = 0.03). Similarly, anti-CCP titers were significantly elevated in fatal patients relative to those achieving clinical remission (*P* = 0.04). While bacteremia incidence trended lower in the survivor group (11.1% [1/9] vs. 75.0% [3/4] in fatal cases), this difference lacked statistical significance (*P >*0.05). Furthermore, no statistically significant differences in gender, underlying medical condition, RA disease activity, RA disease duration, multi-organ involvement, pre-onset treatment, RF levels, or histopathological findings were observed between the two groups (all *P* > 0.05).

**Table 1:** Comparison of demographic and clinical characteristics between the survival and mortality groups.

	Total		Survival		Mortality		Univariate Analysis	
	(n=112		(n=98/110)		(n=12/110)		*P*-value	Odds ratio
	No.	%	No.	%	No.	%		
Age, years								
Range	6-89		6-85		23-89		0.36	
<65 years old	64	57.1	58	59.2	6	50.0		
≥65 years old	45	40.2	37	37.8	6	50.0	0.46	1.57(0.44-5.57)
Not mentioned	3	2.7	3	3.1	0	0.0		
RA history, years								
Range	0-57		0-57		0-20		0.34	
Gender								
Male	36	32.1	29	29.6	7	58.3		
Female	73	65.2	66	67.3	5	41.7	>0.05	0.31(0.10-1.03)
Not mentioned	3	2.7	3	3.1	0	0.0		
Underlying medical condition								
Without an underlying condition	96	85.7	86	87.8	9	75.0		
With underlying conditions	16	14.3	12	12.2	3	25.0	0.21	2.39(0.62-9.58)
RA disease activity								
Active disease	25	22.3	22	22.4	2	16.7		
Clinical remission	87	77.7	76	77.6	10	83.3	>0.99	1.03(0.83-1.18)
Co-infection status								
No co-infection	98	87.5	89	90.8	8	66.7		
Co-infections	14	12.5	9	9.2	4	33.3	0.03	4.94(1.40-20.65)
Bacteraemia	4	28.6	1	11.1	3	75.0		
Others	10	71.4	8	88.9	1	25.0	>0.05	0.04(0.00-0.69)
Organ Involvement in RV								
Single-organ	89	79.5	80	81.6	7	58.3		
Multi-organ involvement	23	20.5	18	8.2	5	41.7	0.06	3.18 (0.98-10.89)
Pre-onset treatment								
Standardized treatment	92	82.1	81	82.7	10	83.3		
Discontinued or untreated	20	17.9	17	17.3	2	16.7	>0.99	0.95(0.19-4.19)
Laboratory finding								
RF, IU/mL [median (p25-p75)]	168.0(88.9-684.3)		163.0(82.9-569.0)	(n=51/58)	507.0(80.01-1472)	(n=8/58)	0.70	
anti-CCP, IU/mL [median (p25-p75)]	200.0(42.8-300.0)		195.6(22.6-300.0)	(n=35/41)	310.5(187.5-569.3)	(n=6/41)	0.04	
Histopathological findings								
Yes	48	42.9	42	42.9	6	50.0		
No	64	57.1	56	57.1	6	50.0	0.64	0.75(0.21-2.65)

RA, rheumatoid arthritis; RF, rheumatoid factor; RV, Rheumatoid vasculitis; anti-CCP, anti-cyclic peptide containing citrulline.

### 3.3 Site of involvement and clinical manifestations

**Figure 2:**
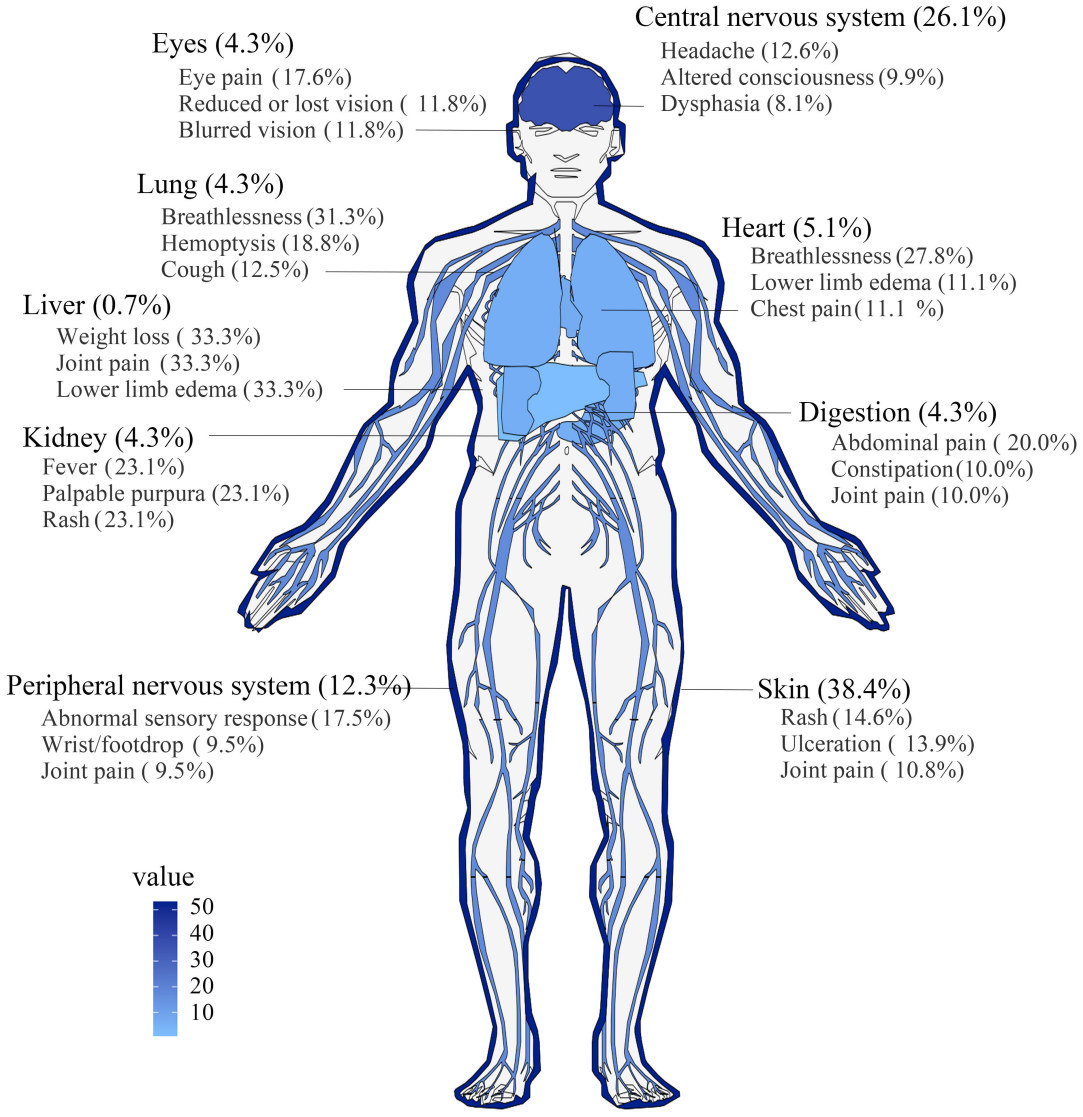
Anatomical distribution mapping of organ involvement in RV. Sites of organ involvement in RV were anatomically mapped using the gganatogram package via R software (version 4.2.3). Darker colors indicated more cases reported.

The case reports we collected demonstrate that RV can affect multiple organ systems, including the skin, nervous system (central and peripheral nervous system), lungs, eyes, heart, kidneys, gastrointestinal tract, and liver. As illustrated in **Figure 2**, cutaneous involvement predominates (38.4%), followed by neurological manifestations (26.1% central nervous system, 12.3% peripheral nervous system), with only one reported case of hepatic vascular involvement. Most presentations exhibited localized symptoms: cutaneous manifestations primarily included rash (14.6%) and ulcers (13.9%); central nervous system involvement manifested as headache (12.6%) and altered consciousness (9.9%); peripheral nervous system involvement predominantly manifests as mononeuritis multiplex presented with limb paresthesia (17.5%) and wrist/foot drop (9.5%). Cardiac involvement predominantly targeted the pericardium (42.9%, 3/7) and pulmonary arteries (57.1%, 4/7), manifesting as dyspnea (27.8%), lower limb edema (11.1%), and chest pain (11.1%). Pulmonary manifestations featured dyspnea (31.3%), hemoptysis (18.8%), and cough (12.5%), while ocular involvement predominantly presented as scleritis (66.7%, 4/6) with associated eye pain (17.6%), blurred vision (11.8%), and visual impairment/loss (11.8%). Among six gastrointestinal cases, mesenteric involvement (83.3%, 5/6) exceeded gastric involvement (16.7%, 1/6), with abdominal pain reported in 20% of patients. Renal and hepatic involvement in RV typically manifests with insidious clinical presentations, primarily characterized by nonspecific systemic symptoms such as fever and weight loss.

**Figure 3:**
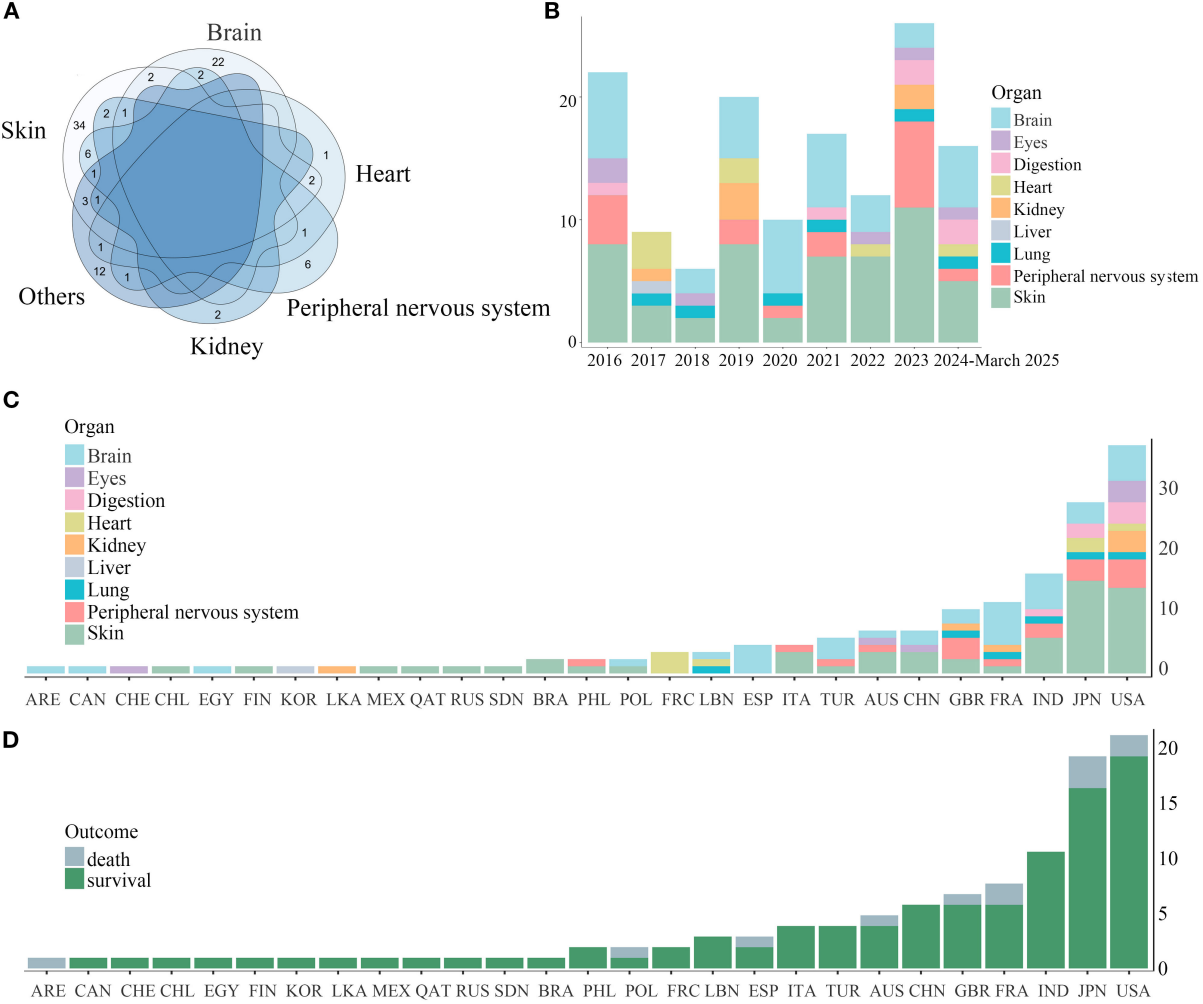
Venn diagram of organ involvement in RV and organ involvement and outcomes of RV across countries (2016-March 2025). **(A)** The overlapping pattern of multi-organ involvement in RV was demonstrated by a Venn diagram. Notably, cutaneous and cerebral involvement typically manifested independently. **(B)** Annual trends of RV organ involvement (2016-March 2025) depicted in stacked areas. **(C)** Country-specific distributions shown in stacked bars. **(D)** Country-specific distribution of survival and mortality cases.

Remarkably, isolated organ involvement was frequent in cutaneous (34/53, 64.2% single-organ cases) and central nervous system (22/36, 61.1% single-organ cases) cases. In contrast, multi-system involvement predominated in cardiac (6/7, 85.7% multi-system cases), peripheral nervous (11/17, 64.7% multi-system cases), and renal (4/6, 66.7% multi-system cases) presentations. Among multi-organ involvement cases, concurrent cutaneous manifestations occurred in 78.3% (18/23) of patients (**Figure 3A**). Over the past decade, reported cases of RV initially experienced a transient decline, followed by a gradual increase (**Figure 3B**). The initial transient decline in RV incidence may reflect improved RA control through earlier biologic DMARD initiation, potentially reducing secondary vasculitis development. Subsequent case escalation likely results might be attributed to advancements in diagnostic techniques and enhanced clinical awareness among physicians regarding RV identification. Regarding the distribution of RV organ involvement, the past two years have seen a modest increase in reports of both gastrointestinal and neurological manifestations. We further analyzed the geographic distribution patterns of RV. The observed predominance of cerebral involvement cases in Spain (ESP) and France (FRA) may reflect inclusion bias from neurovascular case series (**Figure 3C**). After accounting for this confounding factor, RV organ involvement demonstrated no significant geographic specificity. Additionally, country-specific mortality cases did not differ significantly (**Figure 3D**).

### 3.4 Diagnostic and therapeutic modalities

**Figure 4:**
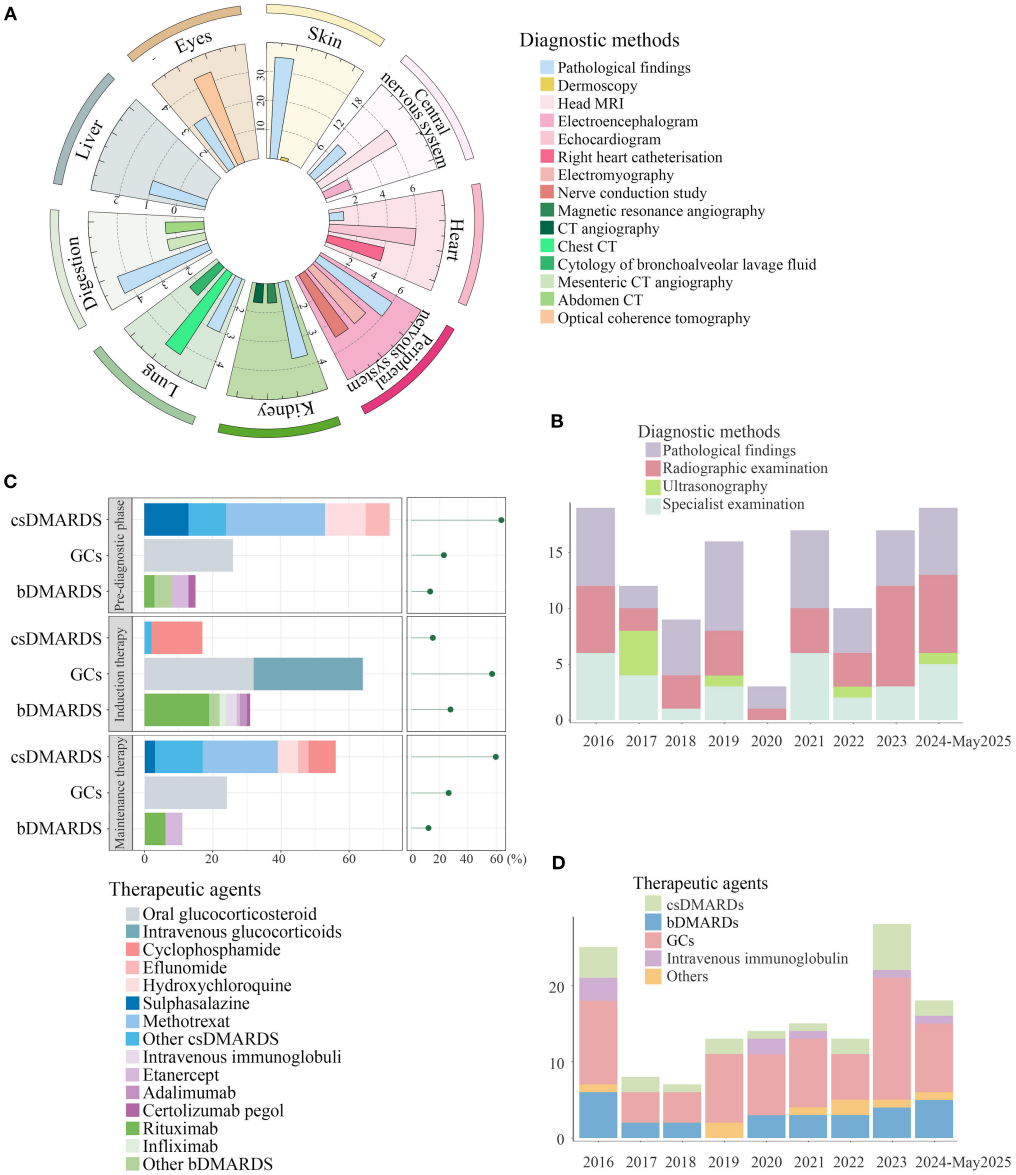
The diagnostic methods and treatment drugs for RV. **(A)** Diagnostic methods for RV across different affected sites were visualized using polar bar plots. Histopathological confirmation was most frequently obtained in cutaneous involvement, while diagnostic methods of other organ systems typically incorporated radiographic examination, ultrasonography, and specialized examinations, including optical coherence tomography, dermoscopy, and electroencephalography. **(B)** Diagnostic approaches for RV from 2026 to May 2025. There has been a slight decline in the utilization of histopathological examinations, while the proportion of imaging studies has increased in the last two years. **(C)** Pharmacological interventions were visualized using combined bar and dot plots, demonstrating the types, quantities, and proportions of therapeutic agents administered during the induction-remission phase, maintenance therapy phase, and pre-RV diagnostic phase. **(D)** Induction therapy for remission in RV from 2026 to May 2025. Over the past decade, bDMARDs showed a characteristic dip followed by a gradual resurgence in clinical application. RV, rheumatoid vasculitis; GCs, glucocorticoids; DMARDs, disease-modifying anti-rheumatic drugs; csDMARDS, conventional synthetic DMARDs; bDMARDS, biological DMARDs.

Among 112 included RV cases, 48 (42.9%) were histopathologically confirmed. Histopathological analysis of skin biopsies revealed perivascular inflammatory cell infiltration as the predominant feature, with coexisting fibrinoid necrosis in 21.9% (7/32), erythrocyte extravasation in 15.6% (5/32), and thrombus formation in 6.3% (2/32). Higher rates of histological confirmation were observed in hepatic (100%, 1/1), gastrointestinal (66.7%, 4/6), renal (66.7%, 4/6), and cutaneous (60.3%, 32/53) involvement (**Figure 4A**). Notably, 43.8% (14/32) of skin biopsies demonstrated leukocytoclastic vasculitis, while renal pathology predominantly exhibited necrotizing or proliferative glomerulonephritis, including two cases with crescents and one with intravascular thrombosis. In addition to histopathological confirmation, dermoscopy supplemented the evaluation of cutaneous RV, magnetic resonance angiography (MRA), and computed tomography angiography (CTA) were utilized for renal involvement assessment, while mesenteric angiography and abdominal CT imaging facilitated gastrointestinal system evaluation. However, histopathological evidence remained scarce in cardiac (14.3%, 1/7), pulmonary (33.3%, 2/6), and cerebral (25.0%, 9/36) cases due to biopsy challenges, prompting reliance on alternative modalities - cranial MRI for CNS involvement, echocardiography for cardiac evaluation, and chest CT for pulmonary assessment. Beyond the aforementioned diagnostic modalities, optical coherence tomography (OCT) was routinely employed for ocular involvement assessment, while electromyography (EMG) and peripheral nerve conduction studies facilitated the diagnosis of mononeuritis multiplex. Our longitudinal analysis of diagnostic approaches for RV over the past decade revealed trends: histological confirmation rates showed a small decline, while imaging utilization increased slightly (**Figure 4B**). As demonstrated in **Figure 4A**, imaging modalities contributed substantially to the diagnosis of systemic involvement, particularly for pulmonary, gastrointestinal, and central nervous system involvements. This observation may explain the recent increase in reported cases with gastrointestinal and neurological involvement over the past two years.

As shown in **Figure 4C**, glucocorticoid therapy (67.9%, including pulse steroid regimens) emerged as the primary induction treatment for RV remission, followed by rituximab (19.6%) and cyclophosphamide (15.2%). During the maintenance phase, conventional synthetic (cs) disease-modifying anti-rheumatic drugs (DMARDs) (40.2%) remained the cornerstone of therapy, with methotrexate (23.2%) and cyclophosphamide (8.0%) being most frequently administered. Surgical interventions were required in cases of gastrointestinal perforation secondary to GI involvement and limb gangrene from cutaneous ischemia, while chronic renal failure necessitated dialysis in renal-compromised patients. Our analysis of treatment trends over the past decade reveals glucocorticoids have consistently remained the primary therapy for inducing RV remission, while bDMARDs showed a characteristic dip followed by a gradual resurgence in clinical application (**Figure 4D**).

### 3.5 Correlation between categories

**Figure 5:**
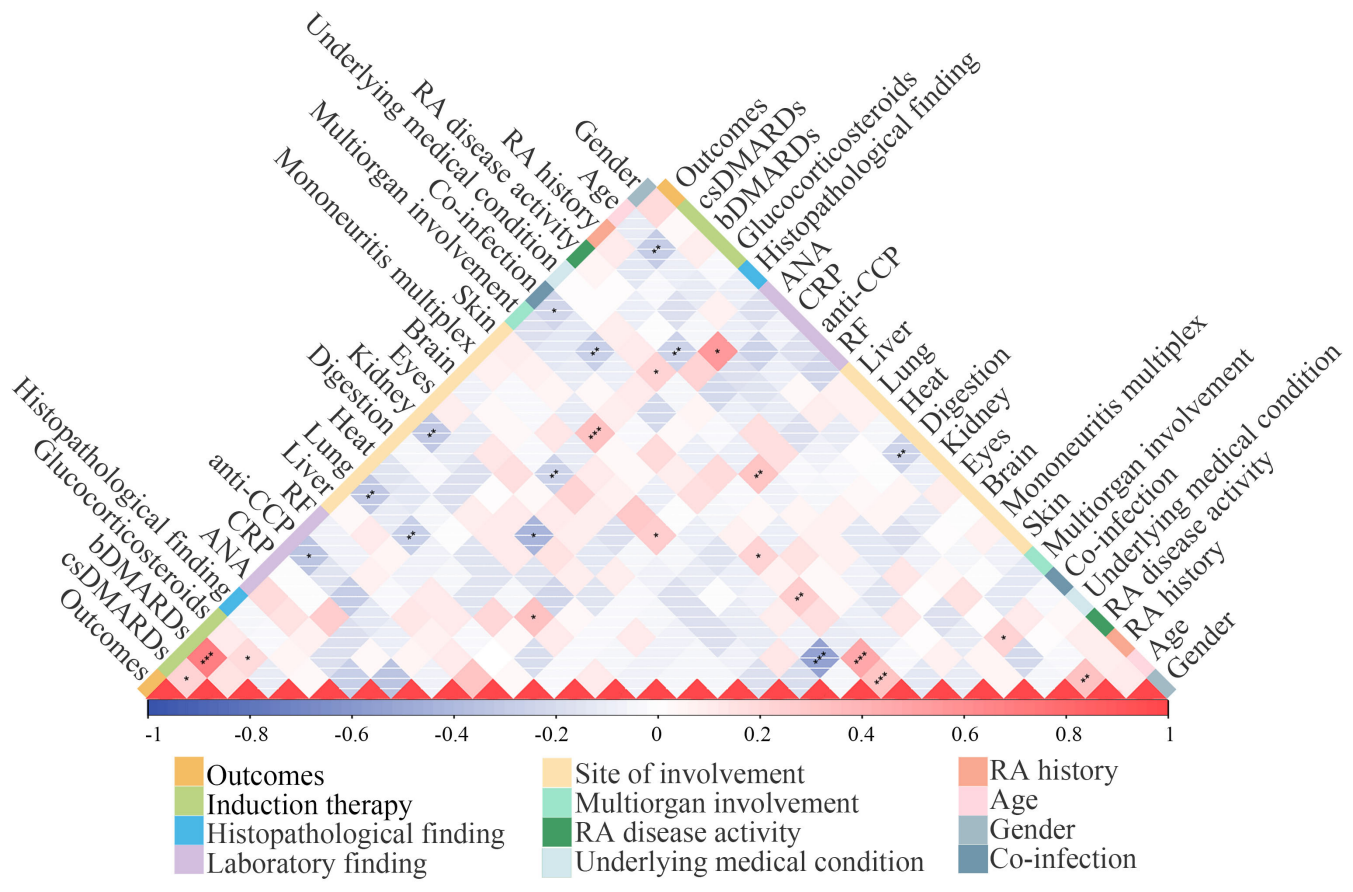
Correlation analysis between categories. The heatmap visualized the association between gender, age, RA history, RA disease activity, underlying medical condition, co-infections, sites of involvement, laboratory findings, histopathological findings, therapeutic modalities, and outcomes. The correlations were measured using the Spearman rank correlation coefficient. Red and blue represented positive correlations and negative correlations, respectively. Color saturation denotes correlation strength. *P < 0.05; **P < 0.01; ***P < 0.001.

We further performed Spearman’s correlation analysis to evaluate associations among gender, age, RA disease duration, RA disease activity, underlying medical condition, co-infection, multi-organ involvement, site of RV involvement, laboratory parameters, histopathological findings, therapeutic medication, and clinical outcomes. Our findings revealed negative correlations with prognosis for co-infection (*P <*0.05), renal (*P <*0.01) and pulmonary involvement (*P <*0.01), and elevated anti-CCP levels (*P <*0.05), whereas csDMARDs (*P <*0.05) and biological (b) DMARDs (*P <*0.001) demonstrated positive prognostic associations (**Figure 5**). Age inversely correlated with csDMARDs during induction therapy (*P <*0.01). Co-infections (*P <*0.01) and pulmonary involvement (*P <*0.01) were negatively associated with bDMARDs. ANA positivity correlated positively with RA disease duration (*P*<0.05) but negatively with renal involvement (*P <*0.05). Anti-CCP levels showed a positive association with cardiac involvement (*P <*0.05), while RF elevation positively correlated with mononeuritis multiplex (*P <*0.05). Moreover, cerebral involvement was inversely associated with cutaneous involvement (*P <*0.001) and histopathological findings (*P <*0.01), whereas peripheral nervous (*P <*0.001) and cutaneous (*P <*0.001), renal (*P <*0.01), and cardiac involvement (*P <*0.05) strongly positively correlated with multi-system involvement.

## 4 Discussion

This systematic analysis of 112 RV case reports from the past decade provides critical insights into the clinical heterogeneity, diagnostic challenges, and prognostic determinants of this rare RA complication. Our findings identify concurrent infections and elevated anti-CCP levels as potential adverse prognostic factors in RV, with significantly higher rates of co-infections and anti-CCP titers observed in fatal cases compared to survivors. These results are in line with Olivé et al’s 2-year follow-up study of 41 RV patients, demonstrating that infection was one of the primary mortality drivers alongside vasculitis progression (4), underscoring the critical prognostic importance of infection management. While previous studies have rarely addressed anti-CCP’s role in RV pathogenesis, it is well-established pathogenic significance in RA synovitis, particularly its superior sensitivity over RF in assessing disease activity and joint destruction (5). Our finding of anti-CCP’s negative correlation with favorable outcomes positions it as a promising Laboratory indicator for RV prognosis.

The exact pathogenesis of RV remains incompletely understood. Existing studies suggest that immune complex deposition—formed by the binding of autoantibodies (rheumatoid factor and anti-CCP antibodies) to their target antigens—plays a central role. These circulating immune complexes deposit on vascular walls, triggering complement cascade activation, upregulating adhesion molecules (e.g., ICAM-1 and VCAM-1), and recruiting neutrophils—ultimately leading to vascular wall inflammation (6, 7). Histopathological examination typically reveals perivascular infiltration of mononuclear cells and/or neutrophils, which might be accompanied by evidence of vascular wall destruction (8). Although RV is a systemic condition, it does not affect all organs. Instead, it predominantly targets small- and medium-sized vessels, with larger arteries (e.g., the aorta) rarely involved. Our analysis reveals predominant cutaneous and central nervous system involvement, typically presenting as isolated organ involvement, contrasting with findings from a 2006 systematic review highlighting cutaneous and peripheral nervous system predominance (9). This might be due to the advancements in neuroimaging techniques and accumulated clinical expertise, enhancing diagnostic sensitivity for CNS vasculitis. Clinically, Cutaneous involvement primarily manifests as small-to-medium vessel vasculitis, presenting with skin eruptions, ulcerations, Bywater lesions, palpable purpura, erythematous patches, painful lesions, Raynaud’s symptoms, and gangrenous complications. Among these manifestations, cutaneous eruptions and ulcerative lesions are the most frequently observed clinical features (10–13). Neurological involvement was observed in a substantial proportion of our study, predominantly manifesting as headache, altered consciousness, dysphasia, postural imbalance, limb weakness/motor dysfunction, seizures, and facial nerve paralysis. These neurologic and motor abnormalities generally responded to corticosteroid and immunotherapy, though neurologic sequelae persisted in select patients (14–16). Notably, cerebrovascular events in RA patients are primarily attributed to comorbid disorders or underlying conditions, with only a minority of cases directly attributable to RV. Peripheral nervous system involvement represents a frequent clinical manifestation of RV, characteristically manifests as mononeuritis multiplex with predilection for distal extremities, clinically presenting with asymmetric sensory disturbances, wrist/foot drop, and motor deficits, though symmetrical neuropathy may occasionally occur (17, 18). Moreover, Moreover, Renal and hepatic involvement pose diagnostic challenges due to their insidious onset, primarily manifesting as non-specific constitutional symptoms such as fever and weight loss (19, 20), underscoring the imperative for heightened clinical vigilance in RV diagnosis.

Currently, there are no formal clinical guidelines for diagnosing RV. Only mononeuritis multiplex and cutaneous vasculitis might be diagnosed without definitive histopathological evidence based on the 1984 diagnostic criteria proposed by Scott and Bacon (2). In our analysis of case reports, over 50% of RV diagnoses lacked pathological evidence, underscoring the reliance on ancillary investigations in most instances. For example, cerebral involvement is assessed through head imaging (MRI/CT) (21), angiography (16), or electroencephalography (14); cardiac manifestations via echocardiography and right heart catheterization (22, 23); and pulmonary lesions using chest CT and bronchoscopy (24). However, even with these modalities, many clinically suspected RV cases remain diagnostically ambiguous without histopathological confirmation, as extra-articular manifestations of RA may originate from vascular/non-vasculitic complications due to diverse etiologies, including infections (e.g., EBV), coexisting immune-mediated disorders (such as systemic lupus erythematosus, Sjögren’s syndrome, or ANCA-associated vasculitis), or underlying comorbidities (e.g., hypertension, diabetes). This diagnostic dilemma is exemplified by Chen’s reported case of RA with urinary obstruction, where RV-related renal involvement was strongly suspected but unverifiable due to the absence of renal biopsy (25).

Glucocorticoid therapy remains the mainstay therapeutic approach for the induction of disease remission in RV. However, our analysis revealed no significant correlation between glucocorticoid use during induction remission and clinical outcomes. Conversely, csDMARDs and bDMARDs, particularly bDMARDs, demonstrated positive prognostic associations, reinforcing emerging evidence for biologics in RV management. Recent years have witnessed expanding clinical applications of biologic agents, including rituximab (26), abatacept (27), and infliximab (28) in the management of rheumatoid vasculitis (RV), with demonstrated favorable clinical efficacy. A systematic review by Alves de Cerqueira et al. (29) demonstrated complete clinical remission in 70% of RV cases following biologic therapy with rituximab or tumor necrosis factor-alpha (TNF-α) inhibitors, but accompanied by a 34% incidence of infection-related adverse events. Our observational data revealed reduced bDMARD use in patients with coexisting infections, highlighting a high level of clinicians’ vigilance towards the heightened risk of infection caused by bDMARDs. Furthermore, during the literature screening phase of this study, we identified multiple cases of RV associated with biologic agents—including adalimumab (30), tocilizumab (31), and golimumab (32)—where clinical improvement typically followed drug discontinuation. This underscores the imperative for heightened vigilance when employing bDMARDs to manage RA, given their paradoxical capacity to induce vasculitis.

Our data reveal a biphasic trend in reported RV incidence over the past decade. The initial decline (2017–2018) may reflect improved RA control through early biologic DMARD intervention, which potentially reduced secondary RV development. The recent rise in reported RV cases appears to reflect two key factors: first, the significantly increased utilization of advanced imaging techniques in clinical practice, leading to improved detection rates; second, and equally important, the growing clinical awareness among physicians which has resulted in more proactive diagnostic approaches - including obtaining targeted tissue biopsies and ordering specialized examinations like nerve conduction studies or contrast-enhanced MR angiography when RV is suspected. Together, technological refinement in diagnostic tools and increased clinician vigilance would collectively enhance early RV detection and facilitate timely therapeutic intervention.

In summary, this systematic investigation significantly advances our understanding of RV by delineating its clinical manifestations, diagnostic challenges, and therapies. A critical finding underscores comorbid infections as a pivotal prognostic determinant in RV, necessitating vigilance throughout disease management. bDMARDs presented a dual role as both therapeutic opportunities and clinical challenges in the management of RV, while capable of ameliorating RV, they might paradoxically induce vasculitis and exacerbate infection risks. Moreover, anti-CCP antibodies as a promising prognostic indicator, warranting validation through prospective multicenter studies to establish their utility in RV outcome prediction.
